# Artificial Intelligence-Supported Evidence Synthesis: A Case Study of Smart Infusion Pump Interoperability

**DOI:** 10.3390/medsci14050533

**Published:** 2026-08-31

**Authors:** Carlos Sanchez-Piedra, Ivo Heyerdahl-Viau, Esther-Elena Garcia-Carpintero, Juan-Manuel Martinez-Nuñez, Francisco-Javier Prado-Galbarro

**Affiliations:** 1 Agency for Health Technology Assessment, Instituto de Salud Carlos III, 28029 Madrid, Spain; carlos.sanchez@isciii.es (C.S.-P.); eegarcia@isciii.es (E.-E.G.-C.); 2 Research Network on Chronicity, Primary Care and Health Promotion (RICAPPS), 20809 Madrid, Spain; 3 Orphan Drugs Laboratory, Department of Biological Systems, Universidad Autónoma Metropolitana Unidad Xochimilco, Mexico City 04960, Mexico; ivoheyerdahl@gmail.com (I.H.-V.); jmartinezn@correo.xoc.uam.mx (J.-M.M.-N.); 4 Research Department, Hospital Infantil de México Federico Gómez, Mexico City 06720, Mexico

**Keywords:** artificial intelligence, large language models, systematic review, smart infusion pumps

## Abstract

Background/Objectives: Artificial intelligence tools have emerged as promising methodological support for systematic reviews and health technology assessment (HTA). Smart infusion pump interoperability represents a relevant case study due to its implications for medication safety, nursing workflow, and hospital quality improvement. The aim was to evaluate the performance of artificial intelligence as a methodological support tool across a systematic review, using the evidence synthesis on smart infusion pump–electronic health record interoperability as a case study. Methods: A systematic review following PRISMA 2020 guidelines was conducted. Searches were performed in MEDLINE, Embase, and Cochrane Library databases. AI-assisted tools (ChatGPT GPT-4 and Open Science Reviewer) were incorporated into data extraction, reporting appraisal based on STROBE criteria, and exploratory identification of methodological limitations under strict human supervision. Concordance between AI-assisted and manual extraction was evaluated descriptively. Results: Overall concordance between AI-assisted and manual data extraction was 82.5% (99/120) across assessed variables. Agreement was highest for structured variables, including study design (10/10; 100.0%), study identification variables (19/20; 95.0%), and participant characteristics (36/40; 90.0%). Agreement was lower for study content variables (18/30; 60.0%) and methodological appraisal (16/20; 80.0%). Among the 21 discrepancies, misclassification errors were most common (13/21; 61.9%), followed by omissions (3/21; 14.3%), incomplete data (3/21; 14.3%), and hallucinations (2/21; 9.5%). AI-assisted identification of methodological limitations showed substantial descriptive agreement with human assessments but demonstrated limited capacity for judgmental interpretations. Conclusions: Artificial intelligence demonstrated utility for structured review tasks such as data extraction and reporting appraisal, but showed limitations in tasks requiring interpretative and methodological judgement. Human oversight therefore remains essential throughout the review process. These findings derive from a single case study and should not be generalised beyond the evaluated context and AI tools.

## 1. Introduction

Smart infusion pumps represent a nursing-critical health technology with direct implications for medication safety, workflow organisation and quality of care. These systems support medication error reduction, which remains among the most persistent safety challenges in hospital practice [1,2], and their implementation has significant consequences for clinical decision-making, workload and patient outcomes [3,4].

Despite a growing body of empirical studies, the evidence base on effectiveness, contextual factors and unintended consequences remains heterogeneous, given the diversity of study designs, outcomes and implementation contexts reported in the literature [3].

The rapid integration of health technologies into clinical practice has intensified the demand for robust and timely evidence to support decision-making in healthcare systems. Health technology assessment (HTA) and systematic reviews are cornerstone methodologies for informing policy. These studies are particularly impactful in nursing, where technology-mediated care delivery, medication safety and workflow optimisation directly affect patient outcomes and professional practice [5,6]. Systematic reviews remain the gold standard for synthesising evidence on effectiveness, safety and implementation of health technologies. However, they are resource-intensive, time-consuming, and vulnerable to delays that may reduce their relevance in fast-evolving areas [7]. They have become increasingly demanding due to the growing volume of biomedical literature, the complexity of contemporary interventions, methodological limitations in primary studies, increasingly sophisticated statistical approaches, and the need to deliver timely evidence to support healthcare decision-making. International reporting standards, including PRISMA 2020, emphasise transparency and methodological rigour, but do not in themselves resolve the structural inefficiencies inherent to manual evidence synthesis [8].

Artificial intelligence (AI) and machine learning methods have emerged as promising methodological support tools to address these challenges. Over the last decade, AI-based approaches have been increasingly applied to key stages of the systematic review process, including citation screening, risk of bias assessment, data extraction, and evidence mapping [9,10]. Active learning algorithms trained on human decisions can substantially reduce screening workload while maintaining high sensitivity, enabling review teams to prioritise clinically relevant evidence more efficiently [11,12]. Tools such as RobotReviewer have demonstrated the feasibility of automating structured risk of bias assessment in randomised trials, highlighting the potential of AI to enhance methodological consistency alongside reviewer judgement [10].

From a health services perspective, the relevance of AI-supported systematic reviews extends beyond technical efficiency. Faster and more scalable evidence synthesis can strengthen the responsiveness of nursing leadership and policymakers to emerging safety concerns, workforce implications and implementation barriers associated with new technologies [13]. Moreover, the integration of AI into evidence synthesis processes aligns with broader digital transformation agendas in nursing, which emphasise the use of advanced analytics to support clinical reasoning, quality improvement and patient-centred care [6,14]. Importantly, AI tools are not intended to replace critical appraisal by clinicians and nurse researchers, but to augment methodological capacity and allow greater focus on interpretation and application of findings within complex care contexts.

Applying AI-supported methodologies to the evaluation of smart infusion pumps provides a concrete opportunity to explore whether these tools can meaningfully support evidence synthesis processes without compromising methodological quality or interpretability. This case study therefore allows for examining both the potential benefits and the practical limitations of integrating AI into systematic review workflows in a clinically relevant domain. A distinctive feature of this study is the integration of AI-assisted support across multiple stages of a systematic review while retaining human reviewers as the final decision-makers. This multi-stage, human-in-the-loop approach provides a more comprehensive assessment of when AI-assisted tools achieve reliable agreement and when human judgement remains essential.

The present study aimed primarily to evaluate the performance of AI as a methodological support tool within a human-led systematic review, using smart infusion pump–electronic health record interoperability as a case study to examine how AI-assisted processes can be integrated into conventional review workflows, assess their contribution to efficiency and rigour, and discuss the implications of these approaches for evidence synthesis and decision-making in healthcare. The primary objective was to assess the concordance between AI-assisted and human data extraction across predefined data elements, using percentage concordance and the classification of discrepancies as the main measures of agreement. Secondary objectives were to examine concordance in reporting-quality assessment and methodological appraisal. We hypothesised that AI-assisted processes would show substantial agreement with human reviewers for structured and explicitly defined tasks, but lower agreement for complex narrative variables requiring contextual or methodological interpretation.

## 2. Materials and Methods

### 2.1. Study Design

This study was conducted as a methodological case study embedded within a systematic review. The systematic review provided a real-world framework for evaluating the performance of AI-assisted tools during selected stages of the evidence synthesis process. The systematic review was focused on evaluating interoperability between smart infusion pumps and electronic health records in hospital settings. The review followed PRISMA 2020 guidelines. This systematic review was conducted in accordance with a prospectively registered protocol in PROSPERO (CRD420261327073), available at https://www.crd.york.ac.uk/PROSPERO/view/CRD420261327073 (accessed on 20 August 2026).

AI was conceptualised as a methodological aid embedded within a conventional, human-led systematic review workflow. All final decisions regarding study inclusion, data interpretation and quality assessment were made exclusively by human reviewers. Accordingly, the principal focus of the present manuscript is the evaluation of AI-assisted review processes rather than the clinical effectiveness of the health technology under review.

### 2.2. Review Question and Eligibility Criteria

The review addressed the following question: What is the effectiveness, safety and implementation impact of interoperability between smart infusion pumps and electronic health record systems in hospital care?

Eligibility criteria were defined a priori using the Population–Intervention–Comparator–Outcome framework. The population included hospitalised patients receiving intravenous medication via infusion pumps. The intervention was interoperability between smart infusion pumps and electronic health records, defined as automated programming, automated documentation or bidirectional data exchange.

Comparators included pre-interoperability periods or non-interoperable workflows.

Outcomes were categorised as primary and additional outcomes according to the registered review protocol. Primary outcomes focused on medication administration safety, including medication errors, patient safety indicators, alert-related outcomes, and safety outcomes associated with interoperability between smart infusion pumps and electronic health records. Additional outcomes included economic, organisational and implementation-related measures, such as documentation quality, workflow indicators, training requirements, usability issues and other process-related outcomes.

Observational studies conducted in real-world hospital settings were included. Simulation studies, conference abstracts, editorials and studies without interoperability were excluded.

### 2.3. Information Sources and Search Strategy

Searches were conducted in MEDLINE (PubMed), Embase and the Cochrane Library, supplemented by reference screening and selected grey literature sources. The final search update was performed in July 2024.

Search strategies combined controlled vocabulary and free-text terms related to smart pumps, electronic health records, interoperability and intravenous medication safety. Searches were limited to English and Spanish. Full strategies are provided in the Supplementary Material.

### 2.4. Study Selection

After duplicate removal, records were screened independently by two reviewers at title/abstract and full-text levels. Disagreements were resolved by consensus. All study selection decisions were made exclusively by human reviewers according to the predefined eligibility criteria.

### 2.5. Use of AI as Methodological Support

The systematic review procedures described above were conducted according to conventional evidence synthesis standards. AI-assisted tools were subsequently incorporated into selected review stages to evaluate their potential methodological contribution. Specifically, ChatGPT (OpenAI) and GPT Open Science Reviewer were used to support data extraction, reporting appraisal based on STROBE criteria, and exploratory identification of methodological limitations. All AI-generated outputs were independently reviewed and validated by the investigators.

ChatGPT was used through the GPT-4 model available at the time of the study. AI-assisted analyses were guided by structured prompts derived from the predefined data extraction template and focused on identifying study characteristics, intervention features, outcome measures, methodological limitations and reporting elements. No model parameters were actively modified, and the default platform settings were used throughout the review process. Outputs generated by both tools were treated as preliminary suggestions and were systematically checked against the original full-text publications before incorporation into the review.

During data extraction, a large-language-model–based tool was employed as a structured reading assistant. The system was prompted to identify predefined data elements, such as study design, intervention characteristics, outcome measures and reported limitations, based on the reviewers’ extraction template. AI-assisted extraction was conducted independently of manual reviewer extraction, and both outputs were subsequently compared. During data extraction and methodological appraisal, a large-language-model–based tool was employed as a structured reading assistant. The system was prompted to identify predefined data elements, such as study design, intervention characteristics, outcome measures and reported limitations, based on the reviewers’ extraction template.

In parallel, all data were extracted manually by a second reviewer. Outputs generated by the AI tool were systematically compared against the manually extracted data, and any discrepancies were reviewed at the full-text level. Any discrepancies between AI-generated outputs and manually extracted data were reviewed against the original full-text publications and resolved by reviewer consensus. Final responsibility for data accuracy remained with the human reviewers.

For reporting quality and methodological appraisal, AI outputs were used exclusively to identify potential reporting gaps and methodological limitations for further reviewer consideration. These outputs did not influence final methodological judgements, which remained based on reviewer assessment and consensus.

At no stage did the AI tool generate independent judgements regarding study quality, relevance or interpretation of findings. Final decisions regarding inclusion, data accuracy, risk of bias and synthesis were made exclusively by the human reviewers.

The prompts used during AI-assisted data extraction and appraisal were developed by members of the review team with expertise in health technology assessment and systematic review methodology. No computer scientists or AI specialists were involved in prompt development. The study did not formally assess learning curves or previous experience with AI-assisted review tools.

### 2.6. Data Extraction

The extraction framework comprised 120 predefined data items grouped into five domains: study identification variables (including authorship, publication year, country), study design characteristics, participant characteristics, study content variables (intervention, outcomes and key findings), and methodological appraisal items. These predefined fields were used for both AI-assisted and manual data extraction.

Data were extracted independently by two reviewers using a standardised template including study design, setting, population, interoperability characteristics and outcomes. AI-assisted outputs were validated line-by-line against original publications. Final responsibility for data accuracy remained with human reviewers.

### 2.7. Risk of Bias and Reporting Quality Assessment

Two reviewers independently assessed risk of bias using ROBINS-I. Reporting quality was evaluated descriptively using STROBE. This assessment was included because the study sought to evaluate the ability of AI-assisted tools to identify reporting strengths and limitations in addition to supporting data extraction.

An AI-based reviewer tool was used as an exploratory aid to identify potential reporting gaps and methodological limitations. AI-generated observations were recorded and subsequently reviewed against the original study reports by the reviewers. These outputs were used to support discussion and critical reflection but did not directly modify ROBINS-I assessments, STROBE evaluations, or final methodological judgements. Final methodological assessments were determined exclusively by human consensus.

### 2.8. Data Synthesis

Due to heterogeneity in study designs and outcomes, meta-analysis was not appropriate. Findings were synthesised narratively using structured tables. Outcomes were grouped into safety-related, process-related and alert-related domains. Interpretation focused on consistency of findings, methodological limitations and implications for clinical practice.

### 2.9. Statistical Analysis

Descriptive and agreement-based analyses were used to evaluate the contribution of AI compared with human reviewers.

Agreement during study selection was assessed using percentage agreement and Cohen’s kappa (κ) with 95% confidence intervals. Discrepancies were classified as over- or under-inclusion and resolved by consensus.

Agreement in data extraction was summarised using percentage concordance across predefined data items. The unit of analysis was the individual extraction item, defined as each predefined data field extracted from each included study. This yielded a total of 120 assessment units across the five included studies. Discrepancies were classified according to their type (omission, incomplete extraction, misclassification, or hallucination). Efficiency of the AI-assisted workflow was evaluated descriptively.

All analyses were designed to assess concordance and methodological support rather than diagnostic accuracy, with the human reviewer as the reference decision-maker.

## 3. Results

### 3.1. Study Selection

A total of 495 records were identified through database searching. After removal of duplicates, 309 records were screened at the title and abstract level by two independent reviewers.

**Figure 1:**
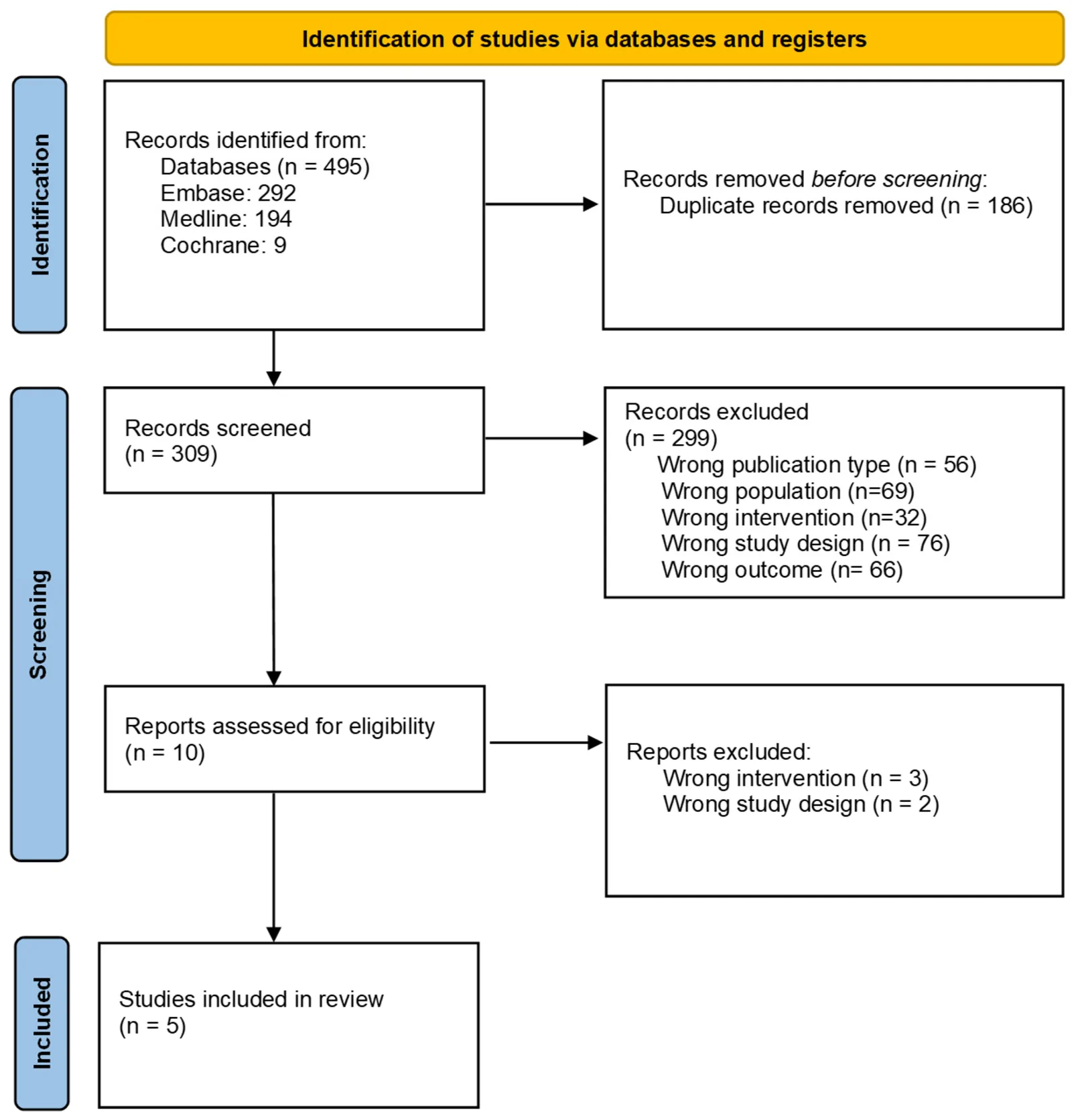
Identification and selection of studies evaluating smart pump–EHR interoperability.

Following title and abstract screening, 10 articles were selected for full-text assessment, of which five met the eligibility criteria and were included in the final synthesis (Figure 1).

The included studies consistently evaluated the impact of smart pump interoperability on medication administration processes and safety-related outcomes. Reported benefits included reductions in selected medication errors, improvements in documentation completeness, increased compliance with drug libraries, and reductions in high-risk alert overrides. However, the evidence remained heterogeneous and was largely based on observational before–after designs, limiting causal inference regarding clinical effectiveness.

### 3.2. Concordance in AI-Assisted Data Extraction

A total of 120 data elements were assessed across the five included studies. Overall concordance between AI-assisted and manual data extraction was 82.5% (99/120) (Table 1).

Agreement was highest for structured variables, including study design (10/10; 100.0%) and study identification variables (19/20; 95.0%). Participant characteristics also showed high concordance (36/40; 90.0%). Lower agreement was observed for study content variables (18/30; 60.0%), which required more complex interpretation of narrative data. Methodological appraisal variables showed moderate agreement (16/20; 80.0%).

Agreement was assessed at the level of individual extraction items. Each predefined data field extracted from each included study constituted one assessment unit, yielding a total of 120 items across the five included studies.

### 3.3. Analysis of Discrepancies in Data Extraction

A total of 21 discrepancies were identified between AI-assisted outputs and manual data extraction (Table 2). Most discrepancies corresponded to misclassification errors, in which the AI inaccurately interpreted extracted information.

Typical misclassification errors involved the oversimplification or incorrect categorisation of study characteristics reported in narrative sections. For example, AI-generated outputs occasionally failed to distinguish between interoperability-related outcomes and broader implementation outcomes, resulting in inaccurate classification of study findings. Hallucination errors were less frequent but typically involved the attribution of methodological details or outcome descriptions that were not explicitly reported in the source article. Although uncommon, such errors could potentially affect the interpretation of study characteristics if not identified through reviewer verification. These findings highlight the importance of maintaining human oversight when using AI-assisted extraction tools, particularly for variables requiring contextual interpretation.

### 3.4. Risk of Bias Assessment and Methodological Appraisal

**Figure 2:**
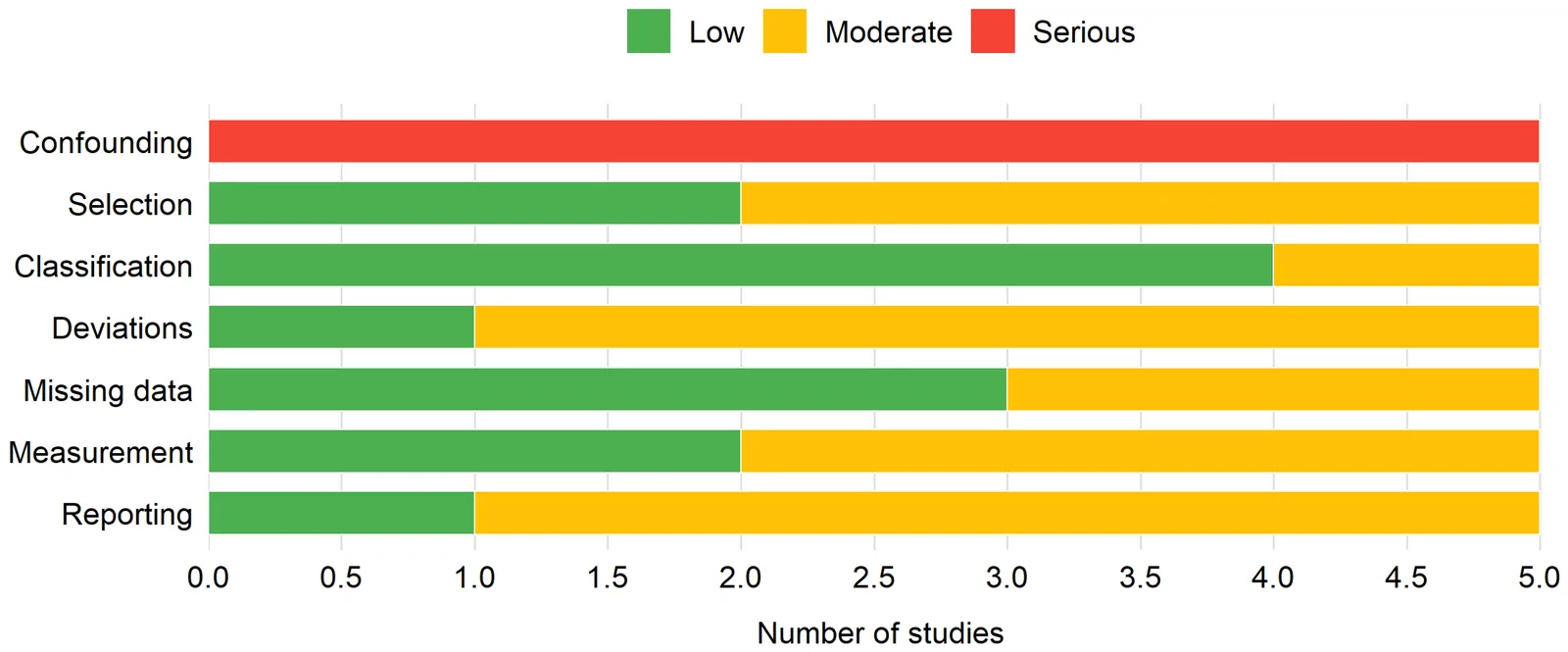
Risk of bias across ROBINS-I domains.

**Figure 3:**
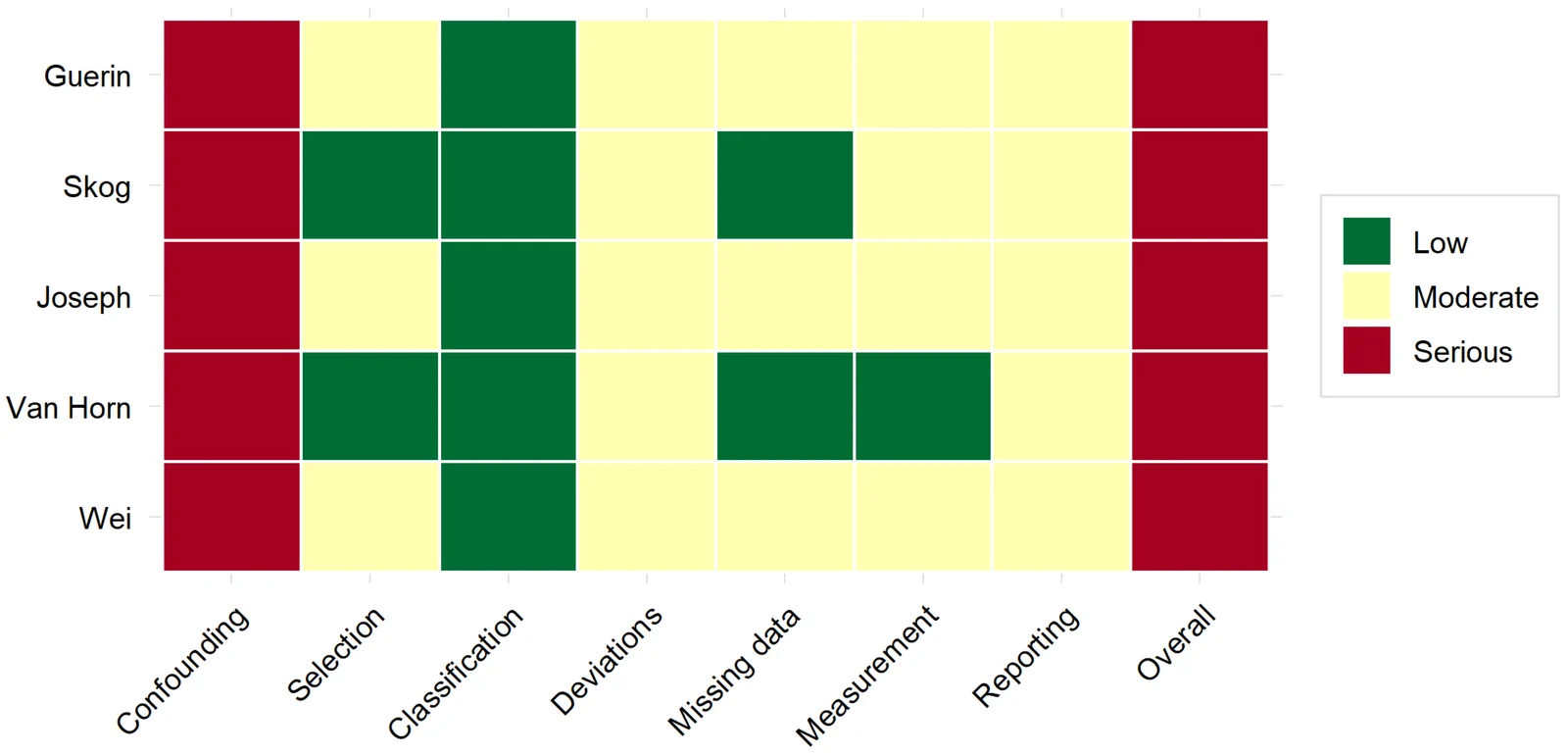
Risk of bias by study and domain.

All included studies used observational before–after designs without a concurrent control group and were therefore evaluated using ROBINS-I. Overall, the studies were judged to have a serious risk of bias, primarily due to the absence of randomisation, lack of a comparator group, and the potential for uncontrolled confounding (Figure 2 and Figure 3).

The domain most consistently affected was confounding, which was rated as a serious risk in all studies. This reflects the inability to adequately control for external or temporal factors that may have influenced the observed outcomes. As a result, changes following implementation of smart infusion pump systems may be partly attributable to factors unrelated to the intervention, such as organisational changes or staff adaptation.

In the remaining domains, including classification of the intervention, selection of participants, measurement of outcomes and management of missing data, the studies were generally assessed as having a low to moderate risk of bias. Interventions were usually well defined, and outcome data were frequently obtained from electronic sources, which may reduce the likelihood of observer bias. However, limitations were identified in the documentation of deviations from intended interventions and in the absence of pre-specified protocols or sensitivity analyses. Overall, all included studies were classified as having a serious risk of bias, primarily driven by uncontrolled confounding and the lack of a control group.

Risk-of-bias assessments presented in Figure 2 and Figure 3 were based exclusively on ROBINS-I evaluations performed by human reviewers. AI-assisted support was considered separately. AI-generated observations were used only to identify potential methodological issues for reviewer consideration and did not contribute to ROBINS-I domain-level judgements.

### 3.5. AI-Supported Assessment of Reporting Quality

Compliance with STROBE reporting recommendations was generally high across the included studies. Most studies clearly described their objectives, populations, interventions and primary outcomes. Nevertheless, recurring limitations were identified, particularly in the reporting of potential confounding factors, the handling of missing data and the justification of sample size.

Multicentre studies tended to show higher levels of reporting completeness, whereas single-centre studies more frequently presented omissions in statistical methods and in the discussion of study limitations. None of the studies reported a pre-published protocol or a pre-specified analysis plan.

AI-assisted completion of the STROBE checklist identified reporting strengths and limitations that were generally similar to those identified by the reviewers. No formal quantitative assessment of agreement was performed.

### 3.6. AI-Supported Identification of Methodological Limitations

AI was used as an exploratory tool to support the methodological appraisal of the included studies through the GPT Open Science Reviewer.

The AI-generated outputs were broadly consistent with the findings obtained using ROBINS-I and STROBE assessments, identifying issues such as the absence of concurrent control groups, the potential influence of observer bias, and limitations in reporting quality. In particular, the AI tool frequently highlighted study characteristics associated with potential confounding, such as before–after designs without concurrent controls, although the final assessment of confounding bias remained exclusively under reviewer judgement.

In addition, the AI tool highlighted methodological strengths in studies with larger sample sizes, clearly defined exclusion criteria, and more detailed analyses of medication error severity.

Overall, while AI-assisted outputs provided a structured overview of methodological features and reporting completeness, they did not replace domain-specific risk-of-bias judgements, which remained based on reviewer assessment.

A comparative overview of the different methodological approaches used in this review, including human and AI-assisted processes, is presented in Table 3.

## 4. Discussion

The integration of AI into evidence synthesis remains an emerging field with limited formal evaluation under transparent methodological conditions; the present study contributes novel evidence regarding both the utility and the limitations of AI tool use during systematic review workflows.

In this study, AI-assisted data extraction demonstrated a high level of agreement with manual methods for structured variables. In terms of methodological appraisal, AI outputs were consistent with human assessments in identifying general limitations and issues related to reporting quality. These results are consistent with a previous study involving 9341 extracted data elements from 63 studies, reporting an overall AI-assisted extraction accuracy of 91.0%, with particularly strong performance for study characteristics and predefined variables [15]. In another study, there was an 80% extraction accuracy using GPT-4 across multiple systematic review domains, with especially good performance for structured PICO-related information such as participants and interventions [16]. Thus, the present findings extend previous feasibility evidence by characterising agreement at the individual data-element level and by identifying the distribution and nature of discrepancies within a real systematic review workflow.

However, a lower concordance was observed for more complex and interpretative elements. Discrepancies were primarily due to misclassification and incomplete extraction, with a small number of hallucination errors also identified. In fact, the identification of hallucination errors should not be overlooked. Although these events had a very low frequency, they still highlight a critical methodological concern regarding the use of generative AI in evidence synthesis. Hallucinations may introduce plausible but inaccurate information into systematic reviews, potentially affecting data interpretation and, therefore, affecting clinical or policy decisions. This highlights the importance of the need for rigorous human verification and line-by-line validation of AI-generated outputs against the original sources. Even though hallucination occurrence may be low, they are a clear characteristic of AI systems [17,18], and, therefore, they are probably one of the principal barriers to the fully autonomous use of AI tools in scientific synthesis workflows.

In this regard, it is important to note that the most important disagreements corresponded to alternative interpretations of complex narrative information. This distinction is important because disagreement between human and AI outputs may sometimes arise from interpretative ambiguity rather than AI fabrication or entirely erroneous output generation. In a recent study, it was concluded that AI’s accuracy in data extraction is predominantly influenced by interpretability rather than hallucination; repeating AI extraction can help identify extraction questions subject to interpretative complexity, enabling refinement before human involvement, suggesting that apparent disagreement may reflect not only hallucination but also ambiguity in how narrative information is interpreted [19].

In contrast to approaches focused primarily on demonstrating whether an AI tool can perform a specific extraction task, our case study integrates AI-assisted prioritisation, extraction, reporting appraisal, and methodological support within a conventional human-led systematic review. This broader workflow allows us to identify both potential advantages and persistent limitations in different tasks. Overall, the present findings emphasise the continued necessity of human oversight throughout the systematic review process; complex tasks such as methodological judgement, interpretation of extracted data, and contextual appraisal require expert judgement that automated systems cannot reliably replicate. Similar conclusions have been reported in recent studies advocating for “human-in-the-loop” models, in which AI enhances reviewer efficiency while supervision and final decisions remain under human responsibility [20,21]. Therefore, AI currently shows limited capacity for domain-specific risk-of-bias evaluation, which requires contextual interpretation and expert judgement. In this context, AI tools should still currently be regarded as a supportive methodological technology, especially when it comes to rapidly identifying variables and study characteristics, rather than a replacement for clinical or methodological expertise.

Despite the active role that humans still need to display in these methodologies, there are plenty of advantages, as the increasingly rapid expansion of medical literature challenges the feasibility of conducting timely systematic reviews using exclusively manual approaches, and delays in evidence synthesis may slow institutional adoption of potentially beneficial medical interventions. In this context, AI-assisted screening and extraction tools may help reduce reviewer workload and accelerate evidence synthesis processes without necessarily compromising methodological quality. For example, a previous study showed that large language models reduced data extraction time by a median of 41 min per study, suggesting that data extraction assisted by AI may offer a viable, more efficient alternative to human-only methods [15].

Undoubtedly, the advantages are solid, and the future looks promising in the field of AI-assisted research. However, this is still a developing approach, and there are still barriers to universally implementing AI tools in clinical evidence synthesis. For example, stand-alone tools should be avoided, and support for more universal and standardised AI platforms is needed to enable transparency, reproducibility, and more collaborative work across fields and regions. Unfortunately, there is still a lack of standard methods for testing and reporting across fields, resulting in evidence that is difficult to track and compare [22]. Another challenge is that outputs generated by AI tools may vary according to prompt design, system updates, and even temperature settings, affecting internal validity and consistency across review processes with a single AI tool [23].

Because of all the previous analysis, we propose that a practical workflow for AI-assisted systematic review may involve AI-tool first-pass extraction from the primary source articles using the predefined fields. AI-generated outputs are treated as provisional rather than final data. This should be followed by independent human verification of all extracted fields against the original source, with particular attention to narrative, contextual, and methodological variables. A dual-reviewer approach may be used for resolving discrepancies identified during AI–human comparison. There should be proper documentation and classification of discrepancies, including omissions, misclassifications, incomplete extraction, and hallucinations. Finally, there should be human resolution and confirmation of all discrepancies before final database locking or synthesis. Only human-verified and corrected information should be incorporated into the final data synthesis. The AI output and the identified discrepancies, corrections, and final decisions should be retained as an audit trail, and could also be useful to study how the AI tool improves over time. This approach would allow AI to reduce repetitive workload while maintaining traceability and methodological accountability.

Regarding the clinical evidence in itself, all included studies carried an observational before–after design, but lacked control groups, resulting in an overall serious risk of bias according to ROBINS-I assessments, limiting causal inference interpretation. This finding reflects the methodological challenges frequently encountered in evaluations of complex healthcare technologies such as infusion pumps. Furthermore, confounding represented the domain most consistently associated with serious risk of bias across all included studies. This is relevant in digital health technologies implemented at the institutional level, where there are multiple methodological challenges inherent to complex hospital-wide technologies, and implementation frequently occurs alongside institutional quality-improvement and staff adaptation.

The findings of the included studies suggest that interoperability between smart infusion pumps and electronic health records may contribute to improvements in medication safety processes, documentation practices, and compliance with drug library safeguards. Nevertheless, the available evidence remains predominantly observational and relies largely on process indicators rather than patient-centred clinical outcomes. An interesting observation from the reporting quality assessment was the higher completeness observed in multicentre studies compared to single-centre research. Multicentre studies may benefit from larger research teams and greater institutional supervision, enhancing reporting transparency and rigour.

This study has some limitations. First, the review included a relatively small number of eligible studies, which may limit the generalisation of the observed concordance patterns between AI-assisted and manual workflows. Therefore, another review with a larger number of studies should be carried out in the future. Second, the methodological evaluation was conducted within a single case-study domain focused on smart infusion pump interoperability, and performance may differ across other healthcare technologies, outcomes or review topics. In addition, no formal assessment of time requirements, workload redistribution, or task completion efficiency was conducted. Therefore, potential efficiency gains associated with AI-assisted workflows should be interpreted cautiously and require confirmation through studies specifically designed to measure operational performance. Finally, the rapid evolution of AI tools means that the performance characteristics observed in this study may soon change substantially as newer systems become available. Therefore, the performance observed in this study should be regarded as a time- and tool-specific estimate rather than a stable characteristic of AI-assisted systematic review processes. The possible association between reporting quality and AI-assisted extraction performance was not formally evaluated and warrants further investigation. One of the main strengths of this study is the evaluation of AI within a real-world systematic review workflow, with a registered PROSPERO protocol, rather than through controlled benchmarking exercises or simulated review scenarios specifically designed to evaluate AI tools. This not only supports transparency and reproducibility, but also provides applied methodological evidence that is directly relevant to clinical and institutional decision-making, enhancing the practical relevance of the findings in complex real-world evidence synthesis scenarios, representative of contemporary healthcare research. Another important strength is the maintenance of rigorous human oversight throughout all stages of the review process, which followed established methodological standards and reporting frameworks, such as PRISMA 2020 recommendations and validated tools such as ROBINS-I for methodological appraisal. Furthermore, the integration of AI within these standardised frameworks not only increases transparency, but also facilitates comparison with previous evidence synthesis studies.

## 5. Conclusions

The present findings suggest that AI has the potential to enhance the efficiency and scalability of selected components of systematic review methodology, particularly for structured tasks such as study selection, data extraction, and reporting appraisal. While AI support appears most appropriate for structured and readily verifiable tasks, narrative and interpretative processes, including data interpretation and contextual appraisal, continue to depend heavily on expert human judgement. Rather than replacing reviewers, AI currently appears best positioned as a supportive methodological technology capable of enhancing efficiency, while remaining subject to continuous and rigorous human validation and oversight. These findings should not be generalised beyond the tools, time, workflow, and smart infusion pump interoperability case study evaluated here.

## Figures and Tables

**Table 1:** Concordance between AI-assisted and manual extraction at the individual data-item level.

Variable	Total Items Assessed	Agreement (n)	Disagreement (n)	Concordance (%)
Study identification variables	20	19	1	95.0
Study design	10	10	0	100.0
Participant characteristics	40	36	4	90.0
Study content variables	30	18	12	60.0
Methodological appraisal	20	16	4	80.0
**Overall**	**120**	**99**	**21**	**82.5**

**Table 2:** Classification of discrepancies in AI-assisted data extraction.

Discrepancy Type	Definition	N Cases	Percentage (%)
Omission	Relevant information not identified by the AI	3	14.3
Incomplete data	Partial extraction of complex variables	3	14.3
Misclassification	Incorrect interpretation of extracted data	13	61.9
Hallucination	Information generated by the AI not present in the source text	2	9.5
**Total**		**21**	**100**

**Table 3:** Comparison of human and AI-assisted methodological processes.

Assessment Component	Human Approach	AI-Assisted Approach	Agreement/Added Value	Key Limitations of AI
Data extraction	Structured manual extraction	Automated extraction of predefined variables	High overall concordance	Errors in complex and narrative variables
Risk of bias (ROBINS-I)	Domain-based expert judgement	No independent AI assessment; reviewer judgement only	Not applicable	Requires contextual interpretation
Reporting quality (STROBE)	Checklist completed by reviewer	Parallel automated checklist completion	High descriptive agreement	Focus on reporting over interpretation
Methodological appraisal	Critical synthesis by reviewers	AI-supported identification of limitations	Consistent with human findings	Limited domain-specific judgement

## Data Availability

No new data were created or analysed in this study. Data sharing is not applicable to this article.

## Supplementary Materials

The following supporting information can be downloaded at: https://www.mdpi.com/article/10.3390/medsci14050533/s1, Table S1: Search strategies; Table S2: PRISMA 2020 Checklist.

## Abbreviations

The following abbreviations are used in this manuscript:

AI	Artificial Intelligence
EHR	Electronic Health Record
GPT	Generative Pre-trained Transformer
HTA	Health Technology Assessment
MEDLINE	Medical Literature Analysis and Retrieval System Online
PICO	Population, Intervention, Comparator, Outcome
PRISMA	Preferred Reporting Items for Systematic Reviews and Meta-Analyses
PROSPERO	International Prospective Register of Systematic Reviews
ROBINS-I	Risk Of Bias In Non-randomised Studies—of Interventions
STROBE	Strengthening the Reporting of Observational Studies in Epidemiology
