# Supplementary material for: Artificial Intelligence-Supported Evidence Synthesis: A Case Study of Smart Infusion Pump Interoperability
Source: Med Sci (Basel). 2026 Aug 31;14(5):533. doi: 10.3390/medsci14050533 (PMC13609719; doi:10.3390/medsci14050533)
Supplement: Supplementary file 1 [file medsci-14-00533-s001.zip › medsci-4492421 Table S1.pdf]

medical sciences

MDPI

Artificial Intelligence-Supported Data Extraction and Appraisal in a Systematic Review: A Case Study of Smart Infusion Pump Interoperability

Supplementary material.

Scheme Strategy

A comprehensive literature search was conducted in MEDLINE (PubMed), EMBASE and the Cochrane Library. Searches were last updated in July 2024 and included publications in English and Spanish. Controlled vocabulary (MeSH and Emtree terms) and free-text keywords related to smart infusion pumps, electronic health record interoperability, autoprogramming, autodocumentation, dose error reduction systems, and intravenous medication errors were combined using Boolean operators. No publication-type restrictions were applied.

The complete search strategies for each database are reproduced below.

**PUBMED Ovid MEDLINE(R) ALL <1946 to July 5, 2024>**		
Nº	Query	Resultados
#1	smart NEAR/2 pump*:ti,ab,kw	11606
#2	limit 1 to yr="2014"	194

**EMBASE**		
Nº	Query	Resultados
#1	smart NEAR/2 pump*	523
#2	#2 AND (2014:py OR 2015:py OR 2016:py OR 2017:py OR 2018:py OR 2019:py OR 2020:py OR 2021:py OR 2022:py OR 2023:py OR 2024:py OR 2025:py OR 2026:py)	296

**COCHRANE**		
**Nº**	**Query**	**Resultados**
#1	smart NEAR/2 pump*:ti,ab,kw	9
